# Evaluation of techniques to break seed dormancy in Redroot pigweed (*Amaranthus retroflexus*)

**DOI:** 10.1002/fsn3.3920

**Published:** 2023-12-26

**Authors:** Fatemeh Ahmadnia, Mohammad Taghi Alebrahim, Leyli Nabati Souha, Dana R. MacGregor

**Affiliations:** ^1^ Department of Plant Production and Genetics, Faculty of Agricultural Sciences & Natural Resources University of Mohaghegh Ardabili Ardabil Iran; ^2^ Department of Protecting Crops and the Environment Rothamsted Research Harpenden UK

**Keywords:** dormancy breaking, germination, seed treatments, ultrasonic waves

## Abstract

By identifying the factors that initiate seed dormancy release, we can reliably predict whether a seed will remain dormant within or exit the seed bank and become a seedling. With regard to annual weed species, assessing which factors efficiently break seed dormancy is critical for estimating the number of weed seeds that will develop into problematic weeds. To better understand dormancy breaking in Redroot pigweed (*Amaranthus retroflexus*), dormant seeds were treated with cold stratification (4°C for 30 days), application of gibberellic acid (at 500, 1000, 1500, and 2000 parts per million), ultrasound (for 10, 20, 30, and 40 min), soaking in hot water (90°C for 3, 5, 7, and 10 min), and 98% sulfuric acid (for 1, 2, and 3 min). The results showed that Redroot pigweed seed dormancy was effectively broken by cold stratification, gibberellic acid, and ultrasound. Short treatments with hot water had minimal effect while longer times or treatment with sulfuric acid stopped seed germination. In addition to germination percentage, germination rate, plumule length, radicle length, seedling length, seedling dry weight, and seed vigor index were also measured; similarly, application of gibberellic acid had the most significant effect on these parameters. The results of this study add to our knowledge of what processes effectively or ineffectively break Redroot pigweed seed dormancy and promote growth.

## Graphical abstract

Understanding seed dormancy is critical for understating how many seeds will persist or exit the seed bank. The results showed that Redroot pigweed seed dormancy was effectively broken by cold stratification, gibberellic acid, or ultrasound. Short treatments with hot water had minimal effect while longer times or treatment with sulfuric acid eliminated seed germination.

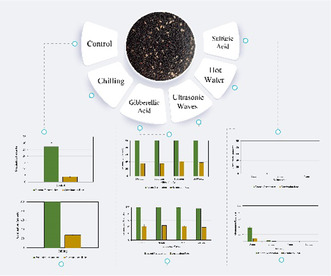

## 1 INTRODUCTION

Seed dormancy is a common phenomenon in many plant species, including agricultural and invasive weeds. Defined as the absence of the embryo‐to‐seedling transition under otherwise favorable conditions, fundamentally seed dormancy allows plants to control when seed germination occurs. The level of dormancy that a seed begins with is influenced by myriad environmental and parental factors (reviewed in Iwasaki et al., 2022). The relative dormancy of seeds after shedding is influenced by various environmental factors that alter seed physiology and behavior; these include soil temperature, water potential, light exposure, fluctuating temperatures, nitrate concentration, soil pH, and the gaseous environment (Travlos et al., 2020). Different species have different dormancy‐breaking requirements which allow them to only germinate, for instance, in the appropriate season (Donohue et al., 2010; Pahlevani et al., 2007) after a specific period of time has passed (Chahtane et al., 2017), or when the seed coat is changed by processes such as subtle ones that alter moisture absorption or leaching of inhibitors or dramatic ones such as passage through the gut of a bird or animal (Penfield, 2017).

Considerable scientific research has been aimed at unraveling the mechanisms behind the establishment, maintenance, and breaking of seed dormancy. This extensive research reflects the pivotal role that dormancy plays in influencing ecological and phenological factors as well as its impact on the agroecosystem. For example, knowing how seed dormancy is maintained or broken is critical to predicting the resilience and persistence of the soil seed bank (Finch‐Savage & Footitt, 2017). With regard to agricultural and invasive weeds, as exit or retention of weed seeds in the soil bank governs population dynamics (Haring et al., 2018) and as the dynamics of weed populations are directly correlated with how much they reduce crop yields and increase agricultural costs (Khakzad et al., 2019; Varah et al., 2020), defining which factors will break weed seed dormancy is important for predicting the impact weeds in the seed bank will have on the crops into which they germinate. Although not always directly applicable to field situations, studying dormancy breaking under laboratory conditions using controlled and standardized methods is useful for defining the dormancy‐breaking requirements of species that are otherwise intractable to study in the field. Furthermore, to conduct laboratory‐based research on germination control and weed establishment, researchers need to break seed dormancy effectively and consistently within a short timeframe. Although various laboratory‐based methods for dormancy breaking have been developed and used, not all of them are effective on all species. Therefore, knowing which methods are most appropriate or inappropriate for a given species or a group of species is important.

Redroot pigweed (*Amaranthus retroflexus*) is a dicotyledonous, monoecious, summer annual weed in the *Amaranthaceae* family. It ranks as the third most prevalent weed worldwide and has spread to 70 tropical and subtropical countries (Costea et al., 2004; Enayati et al., 2019; Holm et al., 1997). This plant reproduces solely through seeds generated from sexual reproduction, with a single plant capable of producing up to 300,000 seeds (Costea et al., 2004; Qi et al., 2017). Accordingly, seed production (Knezevic & Horak, 1998), longevity (Burnside et al., 1996; Gardarin et al., 2010; Taylorson, 1970; Telewski & Zeevaart, 2002; Toole & Brown, 1946), dormancy (Costea et al., 2004; Forcella et al., 1997; Gallagher & Cardina, 1997, 1998), including maternally controlled dormancy (Karimmojeni et al., 2014; Kigel et al., 1977), and germination (Karimmojeni et al., 2014) are important features controlling the population dynamics of Redroot pigweed. As these tiny seeds can remain viable in the soil seed bank for at least 6–10 years (Costea et al., 2004; Jaganathan et al., 2019), measuring dormancy, what breaks it, and germination behavior are critical for defining the seed bank dynamics of Redroot pigweed.

In this study, we measured the germination percentage, germination rate, radicle length, plumule length, seedling length, seedling dry weight, and seed vigor index in response to submergence in cold or hot water, application of gibberellic acid or sulfuric acid (H_2_SO_4_) at various lengths of time or concentrations and compared these to control conditions. We did not consider after‐ripening as this has already been well studied in Redroot pigweed (Enayati et al., 2019; Schonbeck & Egley, 1980, 1981a). These different treatments were chosen not only because they are the standard laboratory methods recommended for breaking seed dormancy and promoting seed germination by the International Seed Testing Association (ISTA, 2018) they also mimic what seeds would experience during changes in temperature in wet soil (cold stratification), alter levels of the hormone that is well known to promote germination (application of gibberellic acid), or methods that variously alter seed coat physiology or permeability (hot water and sulfuric acid). Additionally, we consider ultrasound, which uses sound frequencies in the inaudible range (20–100 kHz) and improves germination rate by stimulating the seed cell wall (Babaei‐Ghaghelestany et al., 2020; Ding et al., 2018; Rifna et al., 2019). These methods have all been successfully used to break the dormancy of other weed species (Alebrahim et al., 2011; Alshallash, 2018; Chadha et al., 2019; Fallahi et al., 2016; Humphries et al., 2018; Kępczyński et al., 2017; Nadjafi et al., 2006; Rahnama‐Ghahfarokhi & Tavakkol‐Afshari, 2007). Our data demonstrate that the application of gibberellic acid, ultrasonication, and cold stratification, most effectively breaks Redroot pigweed seed dormancy. Therefore, we have learned that dormancy breaking in Redroot pigweed requires increases in gibberellic acid or alterations to seed coat physiology that increase moisture absorption and/or leaching of inhibitors.

## 2 MATERIALS AND METHODS

### 2.1 Seed collection and storage

Because this experiment aimed to test the efficacy of the different seed dormancy‐breaking treatments, one large batch of dormant, viable seed was used. This ensured that the outcomes of each treatment could be compared to each other without confounding effects of biological variability that occurs between different seed batches. Redroot pigweed seeds (*Amaranthus retroflexus*) collected from the agricultural fields of Moghan Research and Natural Resources Center (48°20′ E, 38°19′ N) in the month of October year 2021 were used. Plants with ripening inflorescences were collected from the fields and air‐dried at room temperature (27 ± 2) for 3 weeks, and seeds were separated from the inflorescences by rubbing. After separation from the inflorescence, we confirmed that the seeds had high primary dormancy by conducting an initial germination test in distilled water. We also confirmed that these seeds were viable using tetrazolium chloride. The tetrazolium chloride test was performed using the protocol of Esno et al. (1996). In this method, weed seeds were stored in 1% tetrazolium chloride solution for 48 h in the dark at 30°C. We observed the formation of red color around the embryo of most of the seeds tested indicating that a high percentage of the seeds were viable. The seeds used in this experiment were stored in a dry environment at a temperature of 25°C for 6 months between harvest and conducting these experiments. During this time, after‐ripening would have been occurring, releasing some but not all of the primary dormancy of these seeds, leading to the 27.33 ± 3.05% germination rate observed in the control (Table 2).

### 2.2 Experimental design

The experiment was conducted in a completely randomized design (CRD) with three replications. Experimental treatments included a no‐treatment control, cold stratification (4°C), gibberellic acid hormone (500, 1000, 1500, and 2000 ppm), ultrasound (10, 20, 30, and 40 min), sulfuric acid (1, 2, and 3 min), and hot water (3, 5, 7, and 10 min). Seeds from the single seed lot were sterilized by 1% sodium hypochlorite followed by three or more washes in distilled water and randomly placed in 9‐cm‐diameter Petri dishes on No. 42 filter paper. Treatment was then applied, or seeds were left untreated as controls. After treatment or no treatment, three separated technical replicates of 150 healthy seeds were placed on Petri dishes, which were sealed into transparent plastic envelopes and transferred to the seed germinator model BINDER KBW 240 (Germany) and kept in the dark at 25°C for germination. Seeds were monitored for 14 days, and as needed, 10 mL of distilled water was added to each of the Petri dishes to ensure they did not dry out. Germination seeds were counted for 14 days and at one time each day. Seeds with a radicle length of 2 mm were considered germinated seeds (Perry, 1981). Seed growth and vigor traits measurements were done on the 14th day after treatments.

### 2.3 Pretreatment of seed

#### 2.3.1 Cold stratification

To evaluate the cold stratification, the seeds of the Redroot pigweed required for the experiment were placed on a damp (not wet) paper towel. Then, the paper towels were stored in a plastic zipped envelope for 30 days at 4°C in the refrigerator. The seeds were kept uniformly moist throughout the chilling period. The seed storage container was covered entirely and prevented from drying out, and methods were based on Enayati et al. (2019). After 30 days, the seeds were placed on filter paper No. 42 to assess germination rate and seedling parameters.

#### 2.3.2 Application of gibberellic acid

To evaluate the efficacy of the known dormancy‐breaking hormone gibberellic acid (Finkelstein et al., 2008), Redroot pigweed seeds were placed in four concentrations of 500, 1000, 1500, and 2000 ppm for 24 h in the dark at 27 ± 2°C. After the defined time, the seeds were removed from the gibberellic acid container and washed with distilled water based on the protocols in Keshtkar et al. (2009). After the washing process, the seeds were kept at ambient temperature (27 ± 2) for 3 h, and after drying, they were placed on No. 42 filter paper to assess germination rate and seedling parameters.

#### 2.3.3 Application of sulfuric acid

To evaluate the efficacy of sulfuric acid, we followed methods established by Santelmann and Evetts (1971) and Alebrahim et al. (2011). Briefly, the seeds were placed in a sulfuric acid treatment (98% purity) for 1, 2, and 3 min under controlled conditions (under Laboratory hood). After applying the treatment, the seeds were washed with distilled water. After washing, the seeds were kept at ambient temperature (27 ± 2) for 3 h, and after drying, they were placed on No. 42 filter paper for 14 days to assess germination rate and seedling parameters.

#### 2.3.4 Application of ultrasonic waves

To evaluate the efficacy of ultrasound treatment, we used methods developed by Babaei‐Ghaghelestany et al. (2020). Redroot pigweed seeds were exposed to ultrasonic waves for 10, 20, 30, and 40 min to evaluate this treatment. Samples were pretreated by immersing them in an ultrasound bath (a Bandelin DT 255 H model with internal dimensions of 325 × 175 × 305 mm and a volume of 5.5 L). This device can produce ultrasonic waves at a frequency of 35 kHz and a power of 230 W. The ultrasound bath tank was first filled with two liters of distilled water. Then, three replicates of samples were exposed to ultrasound waves with four‐time lengths of 10, 20, 30, and 40 min. After treatment, they were placed on No. 42 filter paper to assess germination rate and seedling parameters.

#### 2.3.5 Treatment in hot water

To evaluate the efficacy of hot water, we used methods described by Majd et al. (2013) and Holm et al. (1997) where Redroot pigweed seeds were placed in a hot water bath (hot water) at 90°C for 3, 5, 7, and 10 min. After the specified times, the seeds were placed at an ambient temperature of 27 ± 2°C for 3 h to dry. After drying, the seeds were placed on No. 42 filter paper to assess germination rate and seedling parameters.

### 2.4 Germination and vigor measurements

All the indicators investigated in this experiment were measured 14 days after the start of incubation at 25°C and dark.

#### 2.4.1 Germination percentage

The seed germination percentage was calculated according to Equation (1) described by Scott et al. (1984), Burnett et al. (2005), and MacGregor et al. (2015).

(1) $$\mathrm{GP}=\frac{S}{T}\times 100$$

where GP is the germination percentage, *S* is the number of germinated seeds, and *T* is the total number of seeds in the experimental sample.

#### 2.4.2 Germination rate

The seed germination rate (GR) is calculated using Equation (2) from Maguire (1962) looking at the number of germinated seeds over time.

(2) $$\mathrm{GR}=\frac{\text{Number of normal seedlings}}{\text{Days to final count}}+\ldots \frac{\text{Number of normal seedlings}}{\text{Days to final count}}$$

#### 2.4.3 Radicle length, plumule, and seedling length

Radicle, plumule, and seedling lengths were measured using a centimeter ruler with an accuracy of 1 mm. Measurements were taken on day 14.

#### 2.4.4 Seedling dry weight

Five samples were taken randomly and placed in the oven at 65°C for 24 h, then weighed on a scale of 0.0001. Measurements were taken on day 14.

#### 2.4.5 Seed vigor index

The seed vigor index was calculated from Equation (3) from Abdul‐Baki and Anderson (1973). Measurements were taken on day 14.

(3) $$V_{i}=\left(\mathrm{PL}+\mathrm{RL}\right)\times \frac{\mathrm{GP}}{100}$$

where *V*_i_ is the seedling vigor, RL is the radicle length (mm), PL is the plumule length (mm), and GP is the germination percentage.

### 2.5 Statistical analysis

All data were analyzed by Rstudio and SAS‐9.4 software. The ANOVA is based on a completely randomized design (CRD) with three replications. Differences among the treatments were evaluated by LSD at level 5%. Correlations between traits were determined using the Pearson's correlation coefficient by R package corrplot in Rstudio software.

## 3 RESULTS

The results shown in Table 1 show that germination percentage, germination rate, plumule length, radicle length and seedling length, seedling dry weight, and seed vigor index of Redroot pigweed seeds were differentially affected by dormancy‐breaking treatments (α ≤ 1%).

**TABLE 1:** Results of analysis of variance for the effect of different methods of break seed dormancy on some germination characteristics of Redroot pigweed seeds.

Source of variation	Degree of freedom	Germination percentage	Germination rate	Radicle length	Plumule length	Seedling length	Seedling dry weight	Seed vigor index
Treatment	16	6888.00**	1110.50**	765.80**	667.57**	2835.07**	2.66**	2940.02**
Error	34	3.68	2.27	3.09	3.03	4.90	0.09	4.98
CV (%)	–	3.38	7.25	8.68	9.12	5.62	25.29	6.30

** Significant at 1% probability level.

### 3.1 Seed germination percentage is promoted by cold, gibberellic acid, ultrasound, and a low dose of sulfuric acid

**FIGURE 1:**
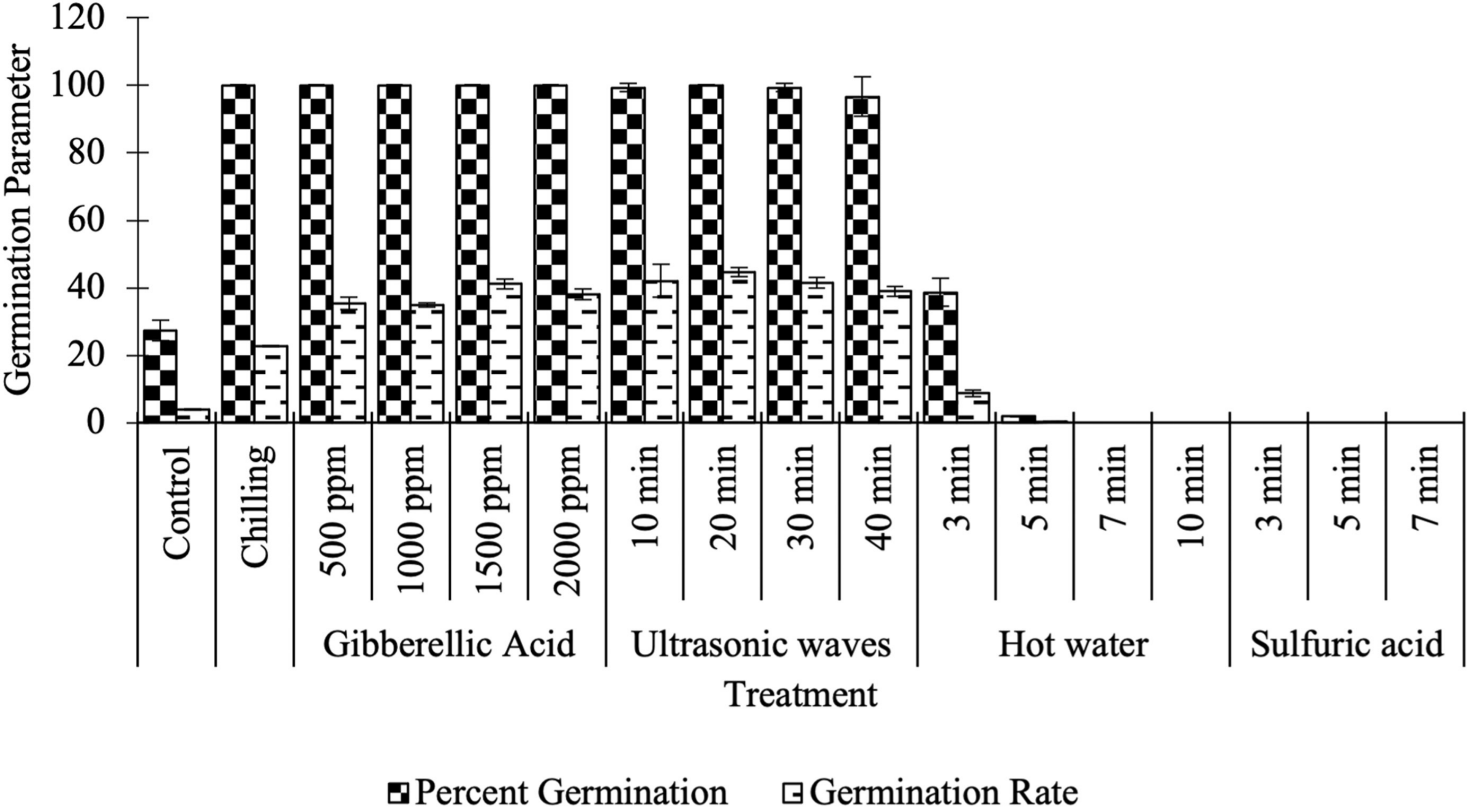
Effect of various treatments on the germination parameters of Redroot pigweed seed monitored for 14 days after no treatment (Control), or treatment by cold stratification (Chilling), application of gibberellic acid at 500, 1000, 1500, and 2000 parts per million, ultrasound treatment for 10, 20, 30, or 40 min, pretreatment with hot water for 3, 5, 7, or 10 min or sulfuric acid for 3, 5, or 7 min. Fifty seeds were used for each technical replicate. Data shown represent averages and standard deviation for three technical replicates for each treatment. Means followed by the same letters do not have a significant difference from each other based LSD multiple range test at the 5% level.

The seed treated with cold stratification, different concentrations of gibberellic acid, and 20 min of ultrasound completely germinated after 14 days (Table 2, Figure 1). Ultrasound for (10, 30, and 40 min) length of time, nearly completely germinated after 14 days; therefore, these treatments are fully able to break the dormancy of Redroot pigweed. Three minutes in hot water was able to promote germination compared to the control; however, longer treatments prevent germination (Table 2, Figure 1). Treatment with sulfuric acid led to no germination of Redroot pigweed seeds (Table 2, Figure 1). N.B. We were unable to assess the other seed germination and vigor parameters for treatments with no germination.

**TABLE 2:** Results of comparing the average germination and some growth components of Redroot pigweed seeds under different treatments of break seed dormancy.

Treatment	GP	GR	RL (mm)	PL (mm)	SL (mm)	SDW (mg)	SVI
*Average ± SD*							
Control	27.33 ± 3.05 d	4.02 ± 0.22 h	24.00 ± 1.73 f	23.00 ± 2.64 f	47.00 ± 1.00 f	1.33 ± 0.33 d	12.82 ± 1.17 h
Chilling	100.00 ± 0.00 a	22.76 ± 0.22 f	32.00 ± 3.00 cd	33.3 ± 3.00 b	65.00 ± 3.00 bc	1.77 ± 0.19 bcd	65.00 ± 3.00 bc
Gibberellic acid							
500 ppm	100.00 ± 0.00 a	35.5 ± 1.8 e	34.33 ± 1.15 b	27.00 ± 1.00 e	61.33 ± 2.08 cd	2.00 ± 0.66 abc	61.33 ± 2.08 cde
1000 ppm	100.00 ± 0.00 a	35.00 ± 0.57 e	32.33 ± 2.51 cd	25.33 ± 1.52 ef	57.66 ± 4.04 de	1.66 ± 0.00 cd	57.66 ± 4.04 ef
1500 ppm	100 ± 0.00 a	41.16 ± 1.42 bc	40.33 ± 0.57 a	38.00 ± 2.00 a	78.33 ± 2.51 a	2.44 ± 0.38 abc	78.33 ± 2.51 a
2000 ppm	100.00 ± 0.00 a	38.11 ± 1.51 d	37.00 ± 2.00 b	30.00 ± 3.6 cd	67.00 ± 2.64 b	2.22 ± 0.5 ab	67.00 ± 2.64 bc
Ultrasonic waves							
10 min	99.33 ± 1.15 ab	42.08 ± 4.83 b	28.00 ± 3.00 e	26.33 ± 2.30 e	54.33 ± 1.52 e	1.78 ± 0.50 bcd	53.98 ± 2.03 f
20 min	100.00 ± 0.00 a	44.66 ± 1.3 a	32.33 ± 2.51 cd	31.33 ± 1.15 bc	63.66 ± 2.88 bc	1.56 ± 0.19 cd	63.66 ± 2.88 bcd
30 min	99.33 ± 1.15 ab	41.57 ± 1.59 b	30.00 ± 0.00 de	32.00 ± 1.00 bc	62.00 ± 1.00 c	2.00 ± 0.33 abc	61.59 ± 1.64 cde
40 min	96.66 ± 5.77 b	38.98 ± 1.45 cd	32.00 ± 2.64 cd	31.33 ± 1.15 bc	63.33 ± 1.52 bc	1.88 ± 0.19 bc	61.26 ± 4.83 cd
Hot water							
3 min	38.66 ± 4.16 c	8.75 ± 0.92 g	22.33 ± 2.51 f	27.33 ± 2.51 de	49.66 ± 5.03 f	1.66 ± 0.33 cd	19.09 ± 1.33 de
5 min	2.00 ± 0.00 e	0.44 ± 0.99 i	0.00 ± 0.00 g	0.00 ± 0.00 g	0.00 ± 0.00 g	0.00 ± 0.00 g	0.00 ± 0.00 i
7 min	0.00 ± 0.00 e	0.00 ± 0.00 i	0.00 ± 0.00 g	0.00 ± 0.00 g	0.00 ± 0.00 g	0.00 ± 0.00 e	0.00 ± 0.00 i
10 min	0.00 ± 0.00 e	0.00 ± 0.00 i	0.00 ± 0.00 g	0.00 ± 0.00 g	0.00 ± 0.00 g	0.00 ± 0.00 e	0.00 ± 0.00 i
Sulfuric acid							
3 min	0.00 ± 0.00 e	0.00 ± 0.00 i	0.00 ± 0.00 g	0.00 ± 0.00 g	0.00 ± 0.00 g	0.00 ± 0.00 e	0.00 ± 0.00 g
5 min	0.00 ± 0.00 e	0.00 ± 0.00 i	0.00 ± 0.00 g	0.00 ± 0.00 g	0.00 ± 0.00 g	0.00 ± 0.00 e	0.00 ± 0.00 i
7 min	0.00 ± 0.00 e	0.00 ± 0.00 i	0.00 ± 0.00 g	0.00 ± 0.00 g	0.00 ± 0.00 g	0.00 ± 0.00 e	0.00 ± 0.00 i
LSD_5%_	3.18	2.5	2.89	2.92	3.67	0.5	3.7
*Fold over Control*							
Control	1.00	1.00	1.00	1.00	1.00	1.00	1.00
Chilling	3.66	5.66	1.33	1.43	1.38	1.33	5.07
Gibberellic acid							
500 ppm	3.66	8.83	1.43	1.17	1.30	1.50	4.78
1000 ppm	3.66	8.71	1.35	1.10	1.23	1.25	4.50
1500 ppm	3.66	10.24	1.68	1.65	1.67	1.83	6.11
2000 ppm	3.66	9.48	1.54	1.30	1.43	1.67	5.23
Ultrasonic waves							
10 min	3.63	10.47	1.17	1.14	1.16	1.34	4.21
20 min	3.66	11.11	1.35	1.36	1.35	1.17	4.97
30 min	3.63	10.34	1.25	1.39	1.32	1.50	4.80
40 min	3.54	9.70	1.33	1.36	1.35	1.41	4.78
Hot water							
3 min	1.41	2.18	0.93	1.19	1.06	1.25	1.49
5 min	0.00	0.00	0.00	0.00	0.00	0.00	0.00
7 min	0.00	0.00	0.00	0.00	0.00	0.00	0.00
10 min	0.00	0.00	0.00	0.00	0.00	0.00	0.00
Sulfuric acid							
3 min	0.00	0.00	0.00	0.00	0.00	0.00	0.00
5 min	0.00	0.00	0.00	0.00	0.00	0.00	0.00
7 min	0.00	0.00	0.00	0.00	0.00	0.00	0.00

*Note*: Means followed by the same letters in the same column do not have a significant difference‐based LSD multiple range test at 5% level.

Abbreviations: GP, Percent germination; GR, Germination rate; PL, Plumule length; RL, radicle length; SDW, Seedling dry weight; SL, Seedling length; SVI, Seed vigor index.

### 3.2 Germination rate

The data measuring germination rates reflect the germination percentages; all treatments that promoted total germination also promoted rate and no rate was able to be measured where no seeds germinated (Table 2, Figure 1). The highest germination rate of Redroot pigweed seeds was obtained after 20 min of ultrasonic treatment (Table 2, Figure 1). Application of gibberellic acid also strongly increased germination rate over control (Table 2, Figure 1). Although chilling treatment and hot water for 3 min increased germination compared to control, the increase was less with these treatments (Table 2).

### 3.3 Radicle length, plumule length, and seedling length and dry weight

**FIGURE 2:**
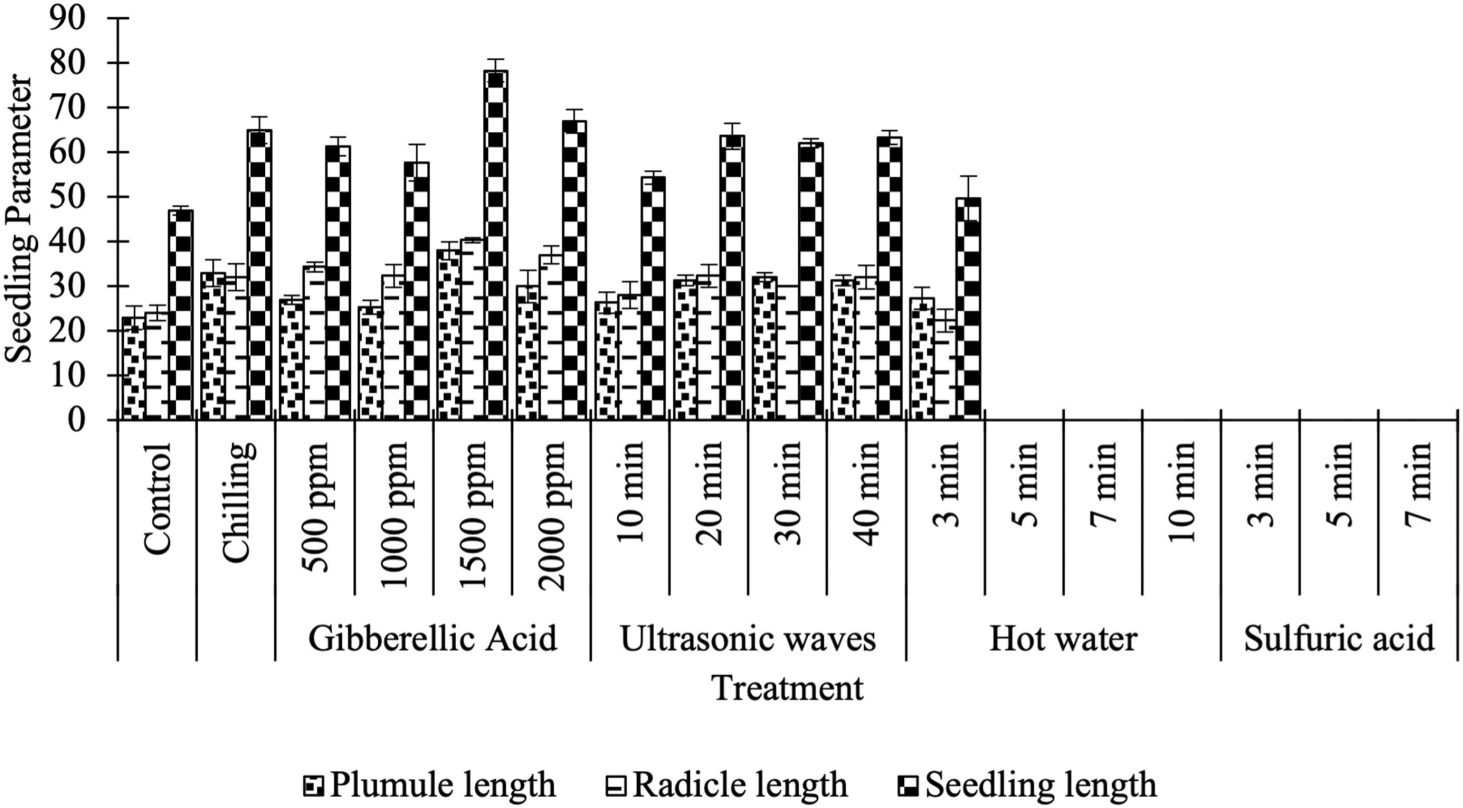
Effect of various treatments on the seedling parameters of plumule length, radicle length, or seedling length (which is the sum of the plumule, seed, and radicle) of Redroot pigweed seed 14 days after no treatment (Control), or treatment by cold stratification (Chilling), application of gibberellic acid at 500, 1000, 1500, and 2000 parts per million, ultrasound treatment for 10, 20, 30, or 40 min, pretreatment with hot water for 3, 5, 7, or 10 min, or sulfuric acid for 3, 5, or 7 min. Fifty seeds were used for each technical replicate. Data shown represent averages and standard deviation for three technical replicates for each treatment. Means followed by the same letters do not have a significant difference from each other based LSD multiple range test at the 5% level.

Application of gibberellic acid 1500 ppm most promoted radicle, plumule, and seedling growth (Figure 2, Table 2). These measurements are also consistent for seedling dry weight (Table 2). Due to the lack of germination in different hot water treatments and sulfuric acid, plumule, radicle, and seedling length in these treatments were unmeasurable. Treatment in hot water for 3 min was similar to control treatment at 22.33 and 24 mm, respectively (Figure 2, Table 2). Compared to the control treatment, storage of seeds in hot water for 3 min reduced the radicle length (22 mm). The control treatment had the shortest plumule length (23 mm) (Table 2). Accordingly, seeds treated with gibberellic acid at 1500 ppm (38 mm), cold stratification, or treatment in hot water (27 mm) but only at 3 min of immersion had longer plumule lengths (Table 2).

### 3.4 Seedling dry weight

Compared to the other parameters, seedling dry weight was less variable between the treatments (Table 2). Consistent with the length measurements, the highest dry weight of Redroot pigweed seedlings was obtained from concentrations of application of gibberellic acid and ultrasound treatment. The control treatment had the lowest seedling dry weight (1.33 mg) after these treatments.

### 3.5 Seed vigor index

Similar to the other parameters, the highest seed vigor index was obtained from gibberellic acid treatment of 1500 ppm (Table 2). The treatments of lower concentrations of gibberellic acid, moisture chilling, ultrasound treatment, and hot water treatment for a short time increased seedling vigor index compared to the control treatment (Table 2). Therefore, seed vigor index is also promoted by these seed pretreatments.

### 3.6 Correlation analysis of measured traits

**FIGURE 3:**
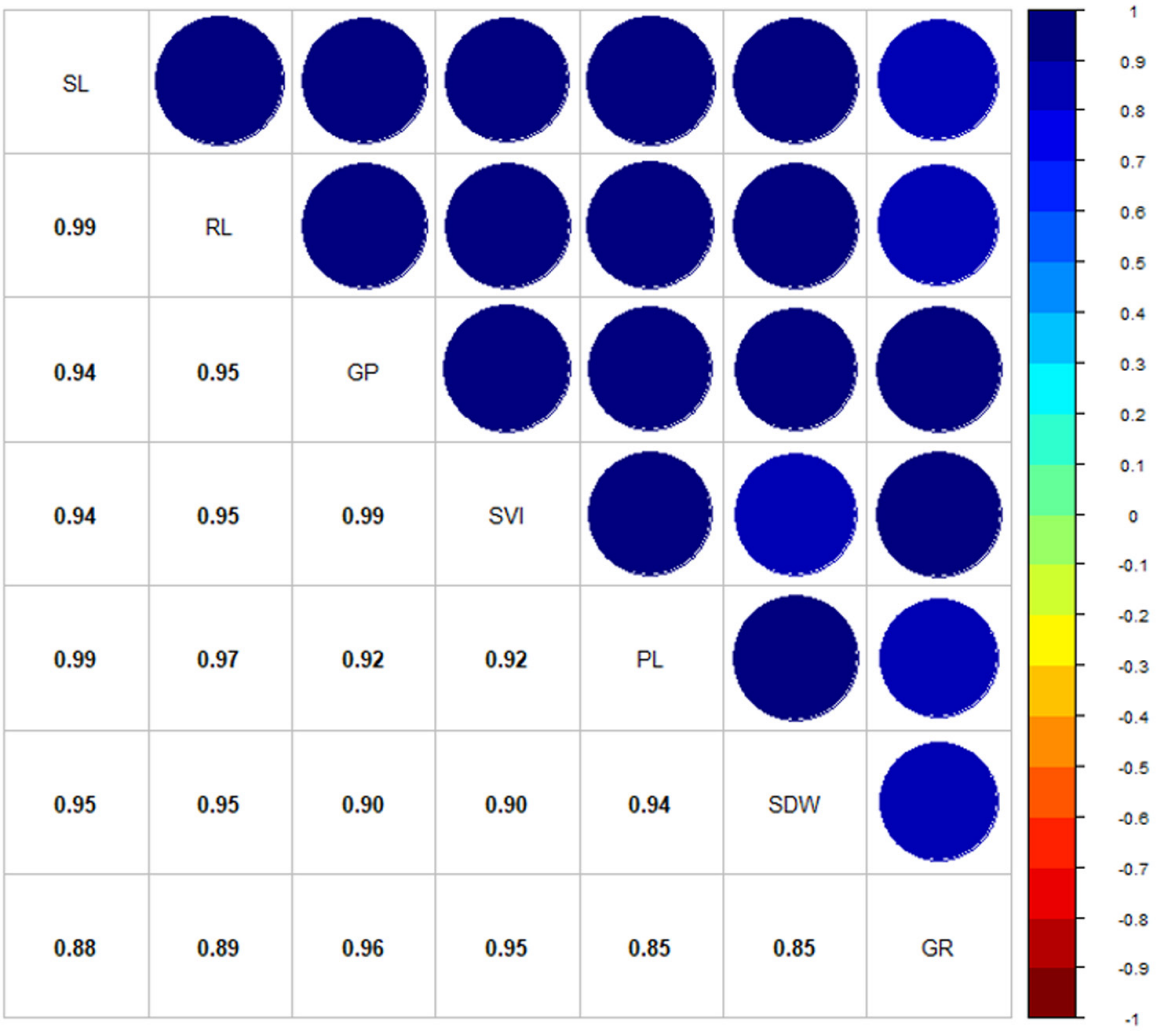
Pearson's correlation coefficients (*n* = 51) among seven quantitative traits on Redroot pigweed. The correlation coefficients were significant at the statistical probability level of 1%. GP, percent germination; GR, germination rate; PL, plumule length; RL, radicle length; SDW, seedling dry weight; SL, seedling length; SVI, seed vigor index.

Pearson's correlation results showed that the examined traits had a positive and significant effect on each other at (*p* ≤ 1%). The results showed that the percentage of seed germination of the Redroot pigweed under the influence of different dormancy treatments had a positive and significant effect on the germination rate (*r* = .963**), plumule length (*r* = .949**), radicle length (*r* = .919**), seedling length (*r* = .942**), seedling dry weight (*r* = .902**), and seed vigor index (*r* = .986**) (Figure 3).

## 4 DISCUSSION

*Amaranthaceae* species are reported to exhibit the most common form of seed dormancy, nondeep physiological dormancy (Baskin & Baskin, 2020). Our data are consistent with Redroot pigweed having nondeep physiological dormancy as the majority of seeds germinated after short periods of chilling stratification or gibberellin treatment, which Baskin and Baskin (2004) define as the key criteria for this type of dormancy. While buried Redroot pigweed seed can remain viable for 6–10 years (Costea et al., 2004), we feel the nondeep physiological classification is appropriate as seeds with deep physiological dormancy are unaffected by the application of gibberellic acid and they frequently require more than 3 months of cold stratification before germination is promoted (Baskin & Baskin, 2004). Moreover, others have reported that Redroot pigweed primary dormancy can be altered by various laboratory processes such as after‐ripening (Enayati et al., 2019; Schonbeck & Egley, 1980, 1981a), incubation at different temperatures (Enayati et al., 2019; Gallagher & Cardina, 1998; Lawrence et al., 2004; Oryokot et al., 1997; Peiguo & Al‐Khatib, 2003; Schonbeck & Egley, 1981a, 1981b), exposure to light with responses relayed via phytochrome‐mediated signaling (Gallagher & Cardina, 1998; Schonbeck & Egley, 1980, 1981b; Taylorson, 1970), treatment with nitrate or nitric oxide, potassium nitrate, ethylene, and gibberellic acid (Gallagher & Cardina, 1998; Kępczyński et al., 2017; Kępczyński & Sznigir, 2013; Rizk et al., 2023; Schonbeck & Egley, 1980, 1981a, 1981b), sulfuric acid (Santelmann & Evetts, 1971) or differences in water potential (Gallagher & Cardina, 1997; Oryokot et al., 1997; Schonbeck & Egley, 1980, 1981b). However, treatments involving acetone, carbon dioxide, sodium hypochlorite, or potassium nitrate were unable to change dormancy in this species (Santelmann & Evetts, 1971; Schonbeck & Egley, 1980).

In this study, we assess different treatments to quantify how efficiently or ineffectively they break Redroot pigweed dormancy. Our data demonstrate that ultrasound treatment can break primary dormancy of Redroot pigweed. Furthermore, we provide evidence of the effects of cold stratification and pretreatments with varying concentrations of gibberellic acid on seedling parameters, including radicle length, plumule length, seedling length, seedling dry weight, and seed vigor index. Finally, our findings demonstrate that pretreatments with either sulfuric acid or hot water are ineffective at breaking Redroot pigweed seed dormancy and in fact, reduce germination from 27% to 0%.

Our data revealed that both cold stratification and application of more than 500 ppm of gibberellic resulted in the germination of all the examined Redroot pigweed seeds. These findings are consistent with existing data on the effects of gibberellic acid on germination in Redroot pigweed (Rizk et al., 2023). The efficacy of cold stratification and exogenous gibberellic acid for breaking dormancy is well documented in various plant species (Finch‐Savage & Leubner‐Metzger, 2006; Holdsworth et al., 2008). For instance, previous research has extensively explored the molecular and physical changes that occur in response to gibberellic acid levels, as well as the effects of after‐ripening and imbibition on dormancy (Finch‐Savage & Leubner‐Metzger, 2006; Holdsworth et al., 2008). These signaling pathways are interconnected, as both cold and light promote germination through increased expression of specific biosynthetic genes in the gibberellic acid biosynthesis pathway (reviewed in Finch‐Savage & Leubner‐Metzger, 2006). Although many or all of these processes could be affected in Redroot pigweed seeds by exogenous gibberellic acid, further research is needed to determine the precise mechanisms by which gibberellic acid is altering seed dormancy in this species.

Our data show that pretreating seeds with ultrasound effectively promoted germination percentage and rate (Figure 1, Table 2). This finding is consistent with previous studies in other species that have utilized ultrasound to promote seed germination (Babaei‐Ghaghelestany et al., 2020; Ding et al., 2018; Kratovalieva et al., 2012; Miano et al., 2015; Rifna et al., 2019). For example, Ding et al. (2018) investigated various physicochemical properties of dehulled rice and showed that ultrasound treatment altered the surface microstructure of rice seeds as well as differences in amylase activity and energy use during germination. Similarly, Babaei‐Ghaghelestany et al. (2020) explored the use of ultrasound to promote germination in *Chenopodium album* and reported increases in germination percentage, seedling dry weight, and measures of seedling vigor. Ultrasound treatments are believed to induce changes in the seed coat, facilitating influx or efflux of water and mineral elements into cells (Miano et al., 2015; Qin et al., 2012) and/or to increase the activity of alpha‐amylase enzyme and therefore the rate of starch metabolism (Yaldagard et al., 2007, 2008). Again, further work is needed to determine the precise mechanism by which ultrasound promotes Redroot pigweed germination and seedling vigor.

Similar to ultrasound, the application of sulfuric acid can damage the seed coat and sclerosing cells allowing water to penetrate and alleviating dormancy caused by the lack of water influx (Baskin & Baskin, 2014; Santelmann & Evetts, 1971). This method has been successfully used to break weed seed dormancy in various species, including in *Acroptilon repens* (Alebrahim et al., 2011), *Astragalus gossypinus* Fisher. (Mehrabi & Hajinia, 2019), as well as various grass seeds (Burton, 1939), forage legumes (Kimura & Islam, 2012), or *Vigna* species (Wang et al., 2007). The duration of sulfuric acid application can vary across studies, with different treatment times promoting germination to varying degrees, typically ranging from 1 to 20 min. However, in our study, we found that any length of pretreatment with sulfuric acid significantly reduced seed germination, suggesting that the treatment led to seed mortality. Acid scarification is typically effective in species with hard seed coats, such as forage legumes (Kimura & Islam, 2012). Redroot pigweed is reported to have a tough seed coat, and although photoperiod, temperature, or level of solar radiation experienced by the parent plant leads to changes in germinability, germination percentage was not correlated to the thickness of Redroot pigweed seed coats (Kigel et al., 1977). It is not clear whether these maternal environmental parameters would alter Redroot pigweed seed coat permeability, as has been seen in Arabidopsis (MacGregor et al., 2015).

In our study, the application of gibberellic acid at a concentration of 1500 ppm had the most significant impact on the measured germination indices, including radicle length, plumule length, seedling length, seedling dry weight, and seed vigor index (Table 2). However, it is not clear whether the observed effects on these parameters were a direct result of the gibberellic acid treatment or simply a consequence of accelerated germination, which provided more time for radicle and plumule growth. The data from Pearson's correlation analysis in Figure 3 show that examined traits had a positive and significant effect on each other. It is possible that exogenous gibberellic acid directly affects cell wall elongation, potentially through altered starch metabolism, affecting the osmotic potential of the cell and facilitating water entry (Arteca, 1996). Another possibility is that gibberellic acid promotes internode elongation, thereby increasing the length of the plumule (Taylor & Wareing, 1979). Like the other measures, further work exploring factors such as cell wall properties, starch metabolism, osmotic potential, and internode elongation is needed to determine if the observed increases in seedling growth are indirect or direct effects of gibberellic acid.

## 5 CONCLUSION

In this study, we investigated methods to break the dormancy of Redroot pigweed (*Amaranthus retroflexus*) seeds. We found that cold stratification and application of gibberellic acid were highly effective in promoting germination, and breaking the dormancy of all seeds examined. Additionally, we show that pretreating seeds with ultrasound also significantly promoted germination percentage and rate. These treatments are known to be useful for breaking dormancy in various plant species. On the other hand, pretreatment with sulfuric acid or hot water, which are known to break dormancy in some plant species, significantly reduced seed germination in Redroot pigweed. Where germination percentage was promoted, we observed correlated alterations in germination indices such as radicle length, plumule length, seedling length, seedling dry weight, and seed vigor index. Further research is needed to determine whether these effects are direct or indirect, such as through changes in cell wall elongation or internode length. Overall, this study provides insights into effective methods for breaking dormancy in Redroot pigweed seeds and highlights the potential of cold stratification, gibberellic acid application, and ultrasound treatment. However, more investigations are required to gain a full understanding of the underlying mechanisms involved in seed dormancy and the specific impacts of these treatments.

## AUTHOR CONTRIBUTIONS

**Fatemeh Ahmadnia:** Conceptualization (equal); data curation (equal); formal analysis (equal); investigation (equal); methodology (equal); resources (equal); writing – original draft (equal); writing – review and editing (equal). **Mohammad Taghi Alebrahim:** Conceptualization (equal); data curation (equal); formal analysis (equal); funding acquisition (equal); methodology (equal); project administration (equal); resources (equal); supervision (equal); validation (equal); writing – original draft (equal); writing – review and editing (equal). **Leyli Nabati Souha:** Conceptualization (equal); data curation (equal); formal analysis (equal); methodology (equal); resources (equal); writing – original draft (equal); writing – review and editing (equal). **Dana R. MacGregor:** Conceptualization (equal); formal analysis (equal); funding acquisition (equal); project administration (equal); supervision (equal); validation (equal); writing – review and editing (equal).

## FUNDING INFORMATION

The study was financially supported by the University of Mohaghegh Ardabili, Iran. Additionally, the research received a grant from Mohammad Taghi Alebrahim (99/د/9128645). Rothamsted Research receives strategic funding from the Biotechnology and Biological Sciences Research Council of the United Kingdom (BBSRC). Dana MacGregor acknowledges support from the Growing Health Institute Strategic Programme [BB/X010953/1; BBS/E/RH/230003A].

## CONFLICT OF INTEREST STATEMENT

The authors declare that they have no competing interests.

## ETHICS STATEMENT

This study does not involve any human or animal testing.

## INFORMED CONSENT

Written informed consent was obtained from all study participants.

## Data Availability

The data that support the findings of this study are available from the corresponding author upon reasonable request.
